# AI-based pathomics model predicts regulatory T cell infiltration and radiotherapy response in IDH-wild-type glioblastoma

**DOI:** 10.3389/fimmu.2026.1817892

**Published:** 2026-05-21

**Authors:** Shaoli Peng, Jialei Chen, Xuezhen Wang, Xingfu Wang, Qiuyuan Yue, Hailin Lan, Jingru Zhang, Yuqi Xie, Meiyan Yang, Jiayu Xiao, Chenyan Guo, Yang Wang, Zanyi Wu, Jinsheng Hong, Mingwei Zhang

**Affiliations:** 1 Department of Radiotherapy, Cancer Center, The First Affiliated Hospital of Fujian Medical University, Fuzhou, China; 2 Key Laboratory of Radiation Biology of Fujian Higher Education Institutions, The First Affiliated Hospital, Fujian Medical University, Fuzhou, China; 3 Department of Pathology, The First Affiliated Hospital of Fujian Medical University, Fuzhou, China; 4 Department of Radiology, Fujian Cancer Hospital and Fujian Medical University Cancer Hospital, Fuzhou, China; 5 School of Medical Imaging, Fujian Medical University, Fuzhou, China; 6 School of Basic Medical Sciences, Fujian Medical University, Fuzhou, China; 7 Department of Radiation Oncology, Huashan Hospital, Fudan University, Shanghai, China; 8 Department of Neurosurgery, The First Affiliated Hospital of Fujian Medical University, Fuzhou, China; 9 Department of Radiotherapy, National Regional Medical Center, Binhai Campus of the First Affiliated Hospital of Fujian Medical University, Fuzhou, China; 10 Department of Oncology, Molecular Oncology Research Institute, The First Affiliated Hospital, Fujian Medical University, Fuzhou, China

**Keywords:** artificial intelligence, glioblastoma, immunotherapy biomarkers, machine learning, pathomics, radiotherapy response, regulatory T cell, tumor microenvironment

## Abstract

**Background:**

Regulatory T cells (Tregs) contribute significantly to immune suppression and therapy resistance in isocitrate dehydrogenase (IDH)-wild-type glioblastoma (GBM), a highly aggressive brain tumor with poor prognosis.

**Methods:**

In this study, we developed an artificial intelligence (AI)-powered pathomics model to predict Treg infiltration and stratify prognosis in GBM patients undergoing radiotherapy. Using high-dimensional features extracted from hematoxylin and eosin-stained biopsies, we constructed a pathomics score (PS) via gradient boosting after feature selection with Minimum Redundancy Maximum Relevance (mRMR) and Relief algorithms.

**Results:**

The model demonstrated strong predictive performance across multi-center cohorts (n > 300), where high PS was significantly associated with elevated Treg levels and reduced overall survival (TCGA: HR = 2.16; validation cohort: HR = 1.706). Gene set enrichment analysis linked high PS to immune-evasive pathways, including Notch and IL-6/JAK/STAT3 signaling, along with increased expression of DNA repair gene RAD50, suggesting a potential association with radiotherapy response.

**Conclusion:**

This AI-based pathomics framework offers a robust and interpretable tool for immunoprofiling and outcome prediction, paving the way for precision radiotherapy and Treg-targeted therapeutic strategies in glioblastoma.

## 1 Introduction

Glioblastoma (GBM) is the most prevalent form of adult glioma, accounting for 57.8% of all primary brain tumors with an incidence of approximately 3.45/100,000 (1). This study applies supervised machine learning, specifically a gradient boosting model, to digital pathology features for immune infiltration prediction and prognostic modeling in glioblastoma. The standard treatment for GBM involves surgical resection whenever possible, followed by postoperative chemoradiotherapy (2). Despite advancements in GBM treatment, such as tumor-treating fields and bevacizumab treatment, the overall survival of patients with GBM remains poor, with a median survival duration of less than 2 years, which can be attributed to the high stemness and pronounced heterogeneity of GBM. Hence, timely diagnosis and effective management of GBM are of paramount importance.

According to the recent World Health Organization classification, GBM refers to the aggressive subtype of glioma characterized by wild-type expression of the isocitrate dehydrogenase (*IDH*) gene, or IDH-wt GBM. Classical prognostic indicators for patients with IDH-wt GBM primarily comprise clinicopathological features, O6-methylguanine-DNA methyltransferase (*MGMT*) promoter methylation, telomerase reverse transcriptase (*TERT*) mutations, and epidermal growth factor receptor (*EGFR*) amplification (3). However, following the promulgation of the CNS 5 criteria in 2021, the population of patients with IDH-wt GBM has shifted, making comprehensive diagnosis and stratified reporting crucial. In particular, the classical prognostic indicators such as *MGMT* (4) and *TERT* (5) have been challenged because of their inadequacy in predicting the treatment response of patients with IDH-wt GBM. Thus, identifying novel biomarkers that can accurately predict prognosis in IDH-wt GBM is of great importance.

The immune microenvironment in GBM is enriched with suppressive cells, including tumor-associated macrophages (TAMs), tumor-associated neutrophils (TANs), regulatory T cells (Tregs), and myeloid-derived suppressor cells (MDSCs), among others, which facilitate tumor growth and worsen prognosis (6). Tregs, in particular, impair T-cell responses and are linked with lower survival rates in GBM patients (7). Studies have shown that an increase in the number or function of Tregs suppresses anticancer immunity (8), promotes tumor progression (9), and is correlated with high recurrence and reduced survival in patients with GBM (10). Presently, targeted therapeutic approaches focusing on Treg, specifically encompassing anti-CD25, anti-CTLA4, and anti-CXCR4 therapies, have advanced into the clinical trial stage (11, 12). Thus, Tregs hold significant prognostic value in IDH-wt GBM and could serve as markers in novel treatment approaches.

Current methods for detecting Tregs, including mRNA sequencing, multicolor cytometry, and multicolor immunofluorescence, are invasive, subjective, and costly, hindering clinical workflow efficiency. Pathomics, a digital analysis of H&E images, offers a rapid, reproducible, and accurate alternative for profiling immune cell infiltration in cancers, enhancing efficiency and cost-effectiveness (13). A study showed machine learning algorithms in melanoma detected immune cell infiltration, linking spatial locations to survival, not just CD8^+^ cell percentages (14). Yu et al. (15) developed a fair-performance image prediction model for cervical cancer immune cell infiltration using risk scores from pathological and immune algorithms (AUC = 0.737). Di et al. (16) demonstrated that a deep-learning model using H&E pathology could predict glioma recurrence and OS, though it lacked specific targets and had limited interpretability. While promising in other cancers, pathomics has not yet been used to evaluate Treg infiltration in GBM.

Recent research indicates that pathomics can link image features with *in situ* immune cell infiltration, including the detection of CD8^+^ cells and tumor-infiltrating lymphocytes (TILs) (14, 17). Additionally, based on our flow cytometry data, we have discerned notable distinctions between Treg cells and other immune cells, particularly in terms of their size, granularity, complexity, and various other attributes. Recognizing the exceptional utility of Hematoxylin and Eosin (H&E) staining for assessing cellular morphology and structure, we propose that an H&E-based pathomics model may serve as a robust predictor of Treg cell infiltration in GBM.

To test this possibility, we aimed to develop a novel pathomics model combined with machine-learning algorithms to predict the level of Treg infiltration in patients with IDH-wt GBM and explore the associated biological processes. Toward this end, we analyzed transcriptome and immunohistochemistry data of patients with IDH-wt GBM from public databases to extract features for building the model with machine-learning algorithms. The infiltration of Tregs in brain tumors was validated using an orthotopic GBM mouse model. We further analyzed the associations of the pathomics model with clinical characteristics and patient prognosis and explored the associated pathways and genes. This study can help to identify new prognostic markers and targets for individualized treatment to improve the outcome for patients with GBM.

## 2 Methods

### 2.1 Patient cohorts and data source

Clinical information of IDH-wt GBM patients was downloaded from The Cancer Genome Atlas (TCGA) database (https://tcga-data.nci.nih.gov/, RRID: SCR_003193), including sex, age at diagnosis, chemotherapy administration, and Karnofsky Performance Scale (KPS) score. The inclusion criteria were as follows: (1) pathologically confirmed GBM, (2) available Treg data, (3) patients who received radiation therapy, (4) access to images of complete pathology sections, and (5) complete clinicopathological information. The exclusion criteria were as follows: (1) poor-quality pathology images (e.g., contamination, blurred images, or blank areas greater than 50%), (2) history of other malignancies, and (3) incomplete follow-up information. Treg infiltration data were obtained from CIBERSORTx (https://cibersortx.stanford.edu/, RRID: SCR_016955).

A retrospective collection of 120 patients with GBM who received radiotherapy from the First Affiliated Hospital of Fujian Medical University was recruited. Inclusion criteria included: (1) age over 18 years, (2) first-time, treatment-naïve GBM patients with histopathological confirmation (3) paraffin-embedded pathological tissues with HE-stained panoramic scanning, and (4) initial intensity-modulated radiotherapy. Exclusion criteria were: (1) recurrent GBM, (2) preoperative local or systemic antitumor therapy, and (3) preoperative hormonal therapy.

This study adhered to the Declaration of Helsinki and received approval from Ethics Committee of First Affiliated Hospital of Fujian Medical University, under the Branch for Medical Research and Clinical Technology Application (approval number: MRCTA, ECFAH of FMU [2022]253).

### 2.2 Pathomics image acquisition, segmentation, and feature extraction

The original H&E-stained immunohistochemistry images of patients with IDH-wt GBM were downloaded from The Cancer Imaging Archive database (https://www.cancerimagingarchive.net/, RRID: SCR_008927).

We combined manual and automatic assessments using the OTSU algorithm. Images were segmented into two parts: the unwanted background and the desired tissue region for the study. Owing to the high resolution of the original pathology slice images, the 40× magnified images were divided into multiple sub-images of 1024 × 1024 pixels, and the 20× magnified images were divided into multiple sub-images of 512 × 512 pixels, which were then upsampled to 1024 × 1024 pixels for analysis. All histopathological images were evaluated under double-blind conditions by two senior pathologists with 10 and 5 years of experience in this field, respectively. Prior to random selection, two senior pathologists manually reviewed all whole-slide images and excluded regions containing necrosis, hemorrhage, staining artifacts, folding, and non-tumor parenchyma. Ten sub-images were then randomly selected exclusively from tumor-rich, histologically representative areas for subsequent feature extraction.

The open-source Pyradiomics package (https://pyradiomics.readthedocs.io/en/latest/) was used to extract 1488 pathomics features from eight major categories from each of the 10 sub-images of every pathology image for each sample, and the average value was used as the pathomics feature for subsequent data analyses. Z-score standardization of the extracted feature values in the training set was performed using the preprocessing function of the R caret package. Samples were randomly divided into training and validation sets at a 7:3 ratio. The mean and standard deviation values of the training set were used to standardize the feature values of the test set.

### 2.3 Machine learning–driven feature selection and predictive modeling

To ensure robust and relevant input for model development, machine learning–based feature selection techniques were employed for pathomics data dimensionality reduction. The minimal redundancy-maximal relevance (mRMR) and relief algorithms were sequentially used for pathomics feature screening and dimensionality reduction. The mRMR algorithm considers the correlation between features, whereas the relief algorithm assigns different weights to the correlation between each feature and category with higher weights indicating stronger classification ability. Both the mRMR and relief algorithms selected the top 20 features, and the intersection of these two sets of features was considered in constructing the model.

For predictive modeling, a gradient boosting machine algorithm was used. This ensemble learning technique builds the final model in a stage-wise manner by iteratively combining multiple weak learners to minimize prediction error. The resulting output, termed the pathomics score (PS), is a continuous composite index derived from the gradient boosting machine model that quantifies the predicted level of Treg infiltration for each patient.

### 2.4 Construction of the GBM orthotopic tumor-bearing mouse model

Six- to eight-week-old female C57BL/6 mice, weighing 18–22 g, were procured from GemPharmatech Co., Ltd., Guangdong, China. The mice were housed in a specific pathogen-free environment at the Laboratory Animal Center of Fujian Medical University. Strict adherence to the relevant ethical guidelines and regulations was followed for all animal experiments and procedures. The animal experimental protocols were approved by the Fujian Medical University (Approval No. IACUC FJMU 2022-0027).

GL261-Luc cells (RRID: CVCL_C9CB) were obtained from Ubigene Biosciences Co., Ltd, Guangdong, China. The cells were cultured in Dulbecco’s Modified Eagle’s Medium (Gibco, USA) supplemented with 1% penicillin–streptomycin and 10% fetal bovine serum at 37°C under 5% CO_2_ condition in an incubator. An orthotopic tumor-bearing mouse model was constructed as a previously described (18). On day 0, a total of 2.5 × 10^7^ GL261-Luc cells were stereotactically implanted into the striatum of the brains of the C57BL/6J mice. Tumor growth was monitored every 3 days using bioluminescence imaging with an IVIS Spectrum Small Animal Imaging System (PerkinElmer, USA). The experimental procedures were initiated in orthotopic tumor-bearing mice when the tumor volumes reached approximately 100 mm^3^.

### 2.5 Flow cytometry

C57BL/6J mice were anesthetized with pentobarbital (50 mg/kg i.p.). GBM tumor tissues were quickly removed, cut into pieces, ground, and centrifuged. Subsequently, approximately 1 × 10^7^ tumor cells were resuspended in phosphate-buffered saline. For cell-surface staining, the cells were blocked with anti-mouse CD16/32 antibody (BD Bioscience, Cat# 553142, RRID: AB_2687830), followed by staining in the dark at 4°C for 30 min using LIVE/DEAD™ Fixable Near-IR Dead Cell Stain Kit (Thermo Fisher Scientific), CD45-Alexa Fluor 700 (BD Bioscience, Cat# 560510, RRID: AB_1102065), CD3-Percp Cyanine 5.5 (BD Biosciences, Cat# 551163, RRID: AB_ AB_394082), CD4-Percp eFluor 710 (Thermo Fisher Scientific, Cat# 46-0041-82, RRID: AB_11150050), CD8-FITC (Thermo Fisher Scientific, Cat# 11-0081-82, RRID: AB_464915), and CD25-PE (Thermo Fisher Scientific, Cat# 12-0251-82, RRID: AB_465607). FoxP3-APC (Thermo Fisher Scientific, Cat# 17-5773-82, RRID: AB_469457) intracellular staining was performed using a FoxP3 staining kit (Thermo Fisher Scientific), according to the manufacturer’s instructions. The percentage of Tregs (DM^-^CD45^+^CD4^+^CD25^+^Foxp3^+^) was determined via flow cytometry (BECKMAN, USA) and analyzed with the Flow Jo software (BD Biosciences).

### 2.6 Pathomics model evaluation

A receiver operating characteristic (ROC) curve was constructed to assess the performance of the prediction model according to the area under the curve (AUC). Additionally, calibration curves were employed to assess the calibration of the predictive model, and the Hosmer–Lemeshow test was utilized to evaluate its goodness of fit. Decision curve analysis (DCA) was used to assess the performance of the model for clinical applications, considering the net benefit of different decision thresholds.

### 2.7 Assessment of PS-related biological processes

The biological significance of the pathomics mechanism estimated by the model was elucidated by analyzing paired pathology images and RNA-sequencing data. The R package “cluster Profiler” was used to perform gene set enrichment analysis (GSEA) of differentially expressed genes in the high and low PS groups. Additionally, we explored the correlation between the PS and the expression of DNA damage repair-related genes, along with the differential expression of immune checkpoint-, inflammation-, and apoptosis-related genes in the high and low PS groups.

### 2.8 Immunofluorescence

Paraffin-embedded sections of brain tissues were dewaxed, hydrated, and treated with EDTA antigen retrieval buffer (pH 8.0) for 20 minutes to repair the antigen. Then, brain tissue sections were blocked with Bovine Serum Albumin (BSA) for 30 minutes. After that, the brain tissue sections were incubated with anti-CD4 (Cat# ab133616, abcam, UK) diluted 1:500 or anti-FOXP3 (Cat# 12653s, Cell Signaling Technology, USA) diluted 1:50 at 4 °C overnight and then with the secondary antibody Alexa Fluor 488(Cat# ab150077, abcam, UK) diluted 1:500 or Cy3 (Cat# ab6939, abcam, Cambridge, UK) diluted 1:200 for 60 minutes, and the nuclei were stained with 4’,6-diamidino-2-phenylindole. After being rinsed with phosphate-buffered saline (PBS), the sections were redyed, sealed, and observed with a 40 × magnification lens on a Zeiss LSM800 confocal microscope.

### 2.9 Statistical analysis

Data analysis was conducted using R software (version 4.1.1). Patients were classified into two Treg infiltration groups—high and low—using the R package “survMisc” to determine the cut-off point. Similarly, the PS of the training set was calculated using the pathomics model, which was merged with the clinical data, and the cut-off point of PS was determined using the “survMisc” package to classify patients into high and low PS groups. This cut-off was selected in the TCGA discovery cohort for exploratory hypothesis generation and was subsequently fixed for validation in the independent FJMU cohort. Categorical variables, expressed as frequencies and percentages, were compared between groups using the χ^2^ test or Fisher’s exact test. Continuous variables are reported as either mean ± standard deviation or median and quartiles. Spearman’s coefficients were calculated to assess the correlation between PS and other covariates. A correlation coefficient greater than 0.3 was defined as a clinically relevant correlation. The Wilcoxon test was used to analyze intergroup variability. Univariate Cox regression analysis and the Kaplan–Meier method were used to assess the clinical prognostic significance of PS in patients with GBM. An interaction test was conducted to investigate interactions between the PS and covariates, such as radiotherapy; a significant interaction test indicates inconsistent results across different stratifications. A sensitivity analysis using an E-value was used to assess the impact of unknown confounders (19, 20). A larger E-value indicates greater robustness of the association to potential unmeasured confounding. Given the balanced class distribution and use of AUC, no imbalance correction was applied. Missing categorical data were handled using dummy variable coding to retain sample size and evaluate missingness impact. All statistical tests were two-sided, and statistical significance was set at a *P*-value less than 0.05.

## 3 Results

### 3.1 Clinical characteristics

The IDH-wt GBM cohort in TCGA database included a total of 195 patients; 108 cases with Treg-related data and superior-quality pathology images were selected for further analysis in this study (**Supplementary Table 1**). The results of multivariate Cox analysis demonstrated that the high Treg-infiltration group (hazard ratio [HR] = 1.556, 95% confidence interval [CI] = 1.007–2.404; *P* = 0.047) was associated with poor prognosis (**Supplementary Figure 1**). Using a 7:3 ratio, the patients were randomly assigned to the training (n = 76) and validation sets (n = 32). No statistically significant differences were observed in the distribution of age, sex, KPS score, or chemotherapy administration between the patients in the training and validation sets (**Table 1**).

**Table 1:** The summary of clinicopathological features of IDH-wt GBM patients in the TCGA cohorts.

Variables	Total (n = 108)	Train (n = 76)	Validation (n = 32)	P value
Treg, n				1
Low	68 (63)	48 (63)	20 (62)	
High	40 (37)	28 (37)	12 (38)	
Age, n				0.702
< 60 years	56 (52)	38 (50)	18 (56)	
≥ 60 years	52 (48)	38 (50)	14 (44)	
Gender, n				0.915
Female	38 (35)	26 (34)	12 (38)	
Male	70 (65)	50 (66)	20 (62)	
KPS, n				0.623
< 80	22 (20)	16 (21)	6 (19)	
≥ 80	59 (55)	43 (57)	16 (50)	
Unknown	27 (25)	17 (22)	10 (31)	
MGMT_promoter, n				0.345
Methylated	33 (31)	26 (34)	7 (22)	
Unknown	25 (23)	18 (24)	7 (22)	
Unmethylated	50 (46)	32 (42)	18 (56)	
TERT_promoter, n				0.3
Mutant	40 (37)	31 (41)	9 (28)	
Unknown	66 (61)	44 (58)	22 (69)	
WT	2 (2)	1 (1)	1 (3)	
Chr_7_gain_Chr_10_loss, n				0.061
Gain chr 7 & loss chr 10	82 (76)	62 (82)	20 (62)	
No combined CNA	26 (24)	14 (18)	12 (38)	
Radiation, n				0.755
NO	20 (19)	13 (17)	7 (22)	
YES	88 (81)	63 (83)	25 (78)	
Chemotherapy, n				0.123
NO	31 (29)	18 (24)	13 (41)	
YES	77 (71)	58 (76)	19 (59)	

### 3.2 Screening and establishment of pathomics features

**Figure 1:**
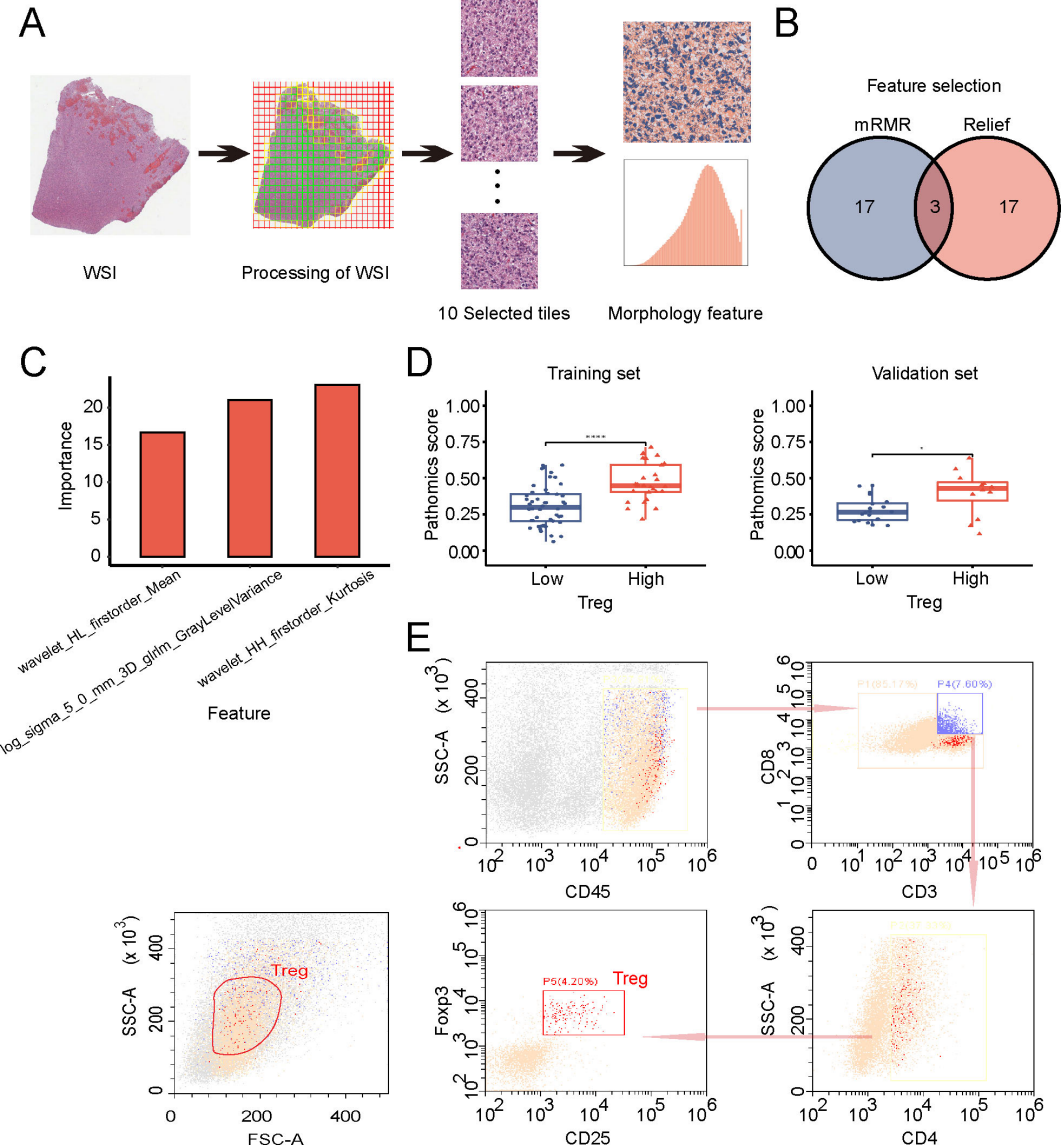
Screening and establishment of pathomics features. **(A)** The workflow of histopathology image processing. **(B)** pathomics feature selection. **(C)** The importance scores of three features extracted in the pathomics model. **(D)** Box plots of pathomics scores (PS) in the high- and low-regulatory T cell (Treg) groups in the training and validation sets. **(E)** Flow cytometry to detect the proportion of Tregs from the tumor tissues of a glioblastoma (GBM) orthotopic mouse model. *P < 0.05, ****P < 0.0001.

The Pyradiomics package was used to extract 1488 features from the pathological images of patients with GBM (**Figure 1A**). The top 20 features were individually selected using the mRMR method and the relief algorithm. The intersection of the selected features from the two algorithms resulted in three features (**Figure 1B**), which were used to construct the pathomics model and calculate the PS based on the gradient boosting machine algorithm. **Figure 1C** displays the importance scores of these three parameters. As shown in **Figure 1D**, the high Treg-infiltration group had a significantly higher PS than did the low Treg-infiltration group in both the training and validation sets (*P* < 0.001). Flow cytometry analysis of the tumor tissues from the mouse orthotopic GBM model showed that Tregs were distinguishable from other immune cells, regardless of their cell size (forward scatter) or granularity (side scatter) (**Figure 1E**). Thus, the PS could serve as a predictive indicator of Treg infiltration. Biologically, a high PS reflects an H&E-based tissue architecture pattern associated with increased Treg infiltration, suggesting a more immunosuppressive and potentially radioresistant tumor state.

### 3.3 Efficacy of the pathomics model to predict the Treg infiltration level

**Figure 2:**
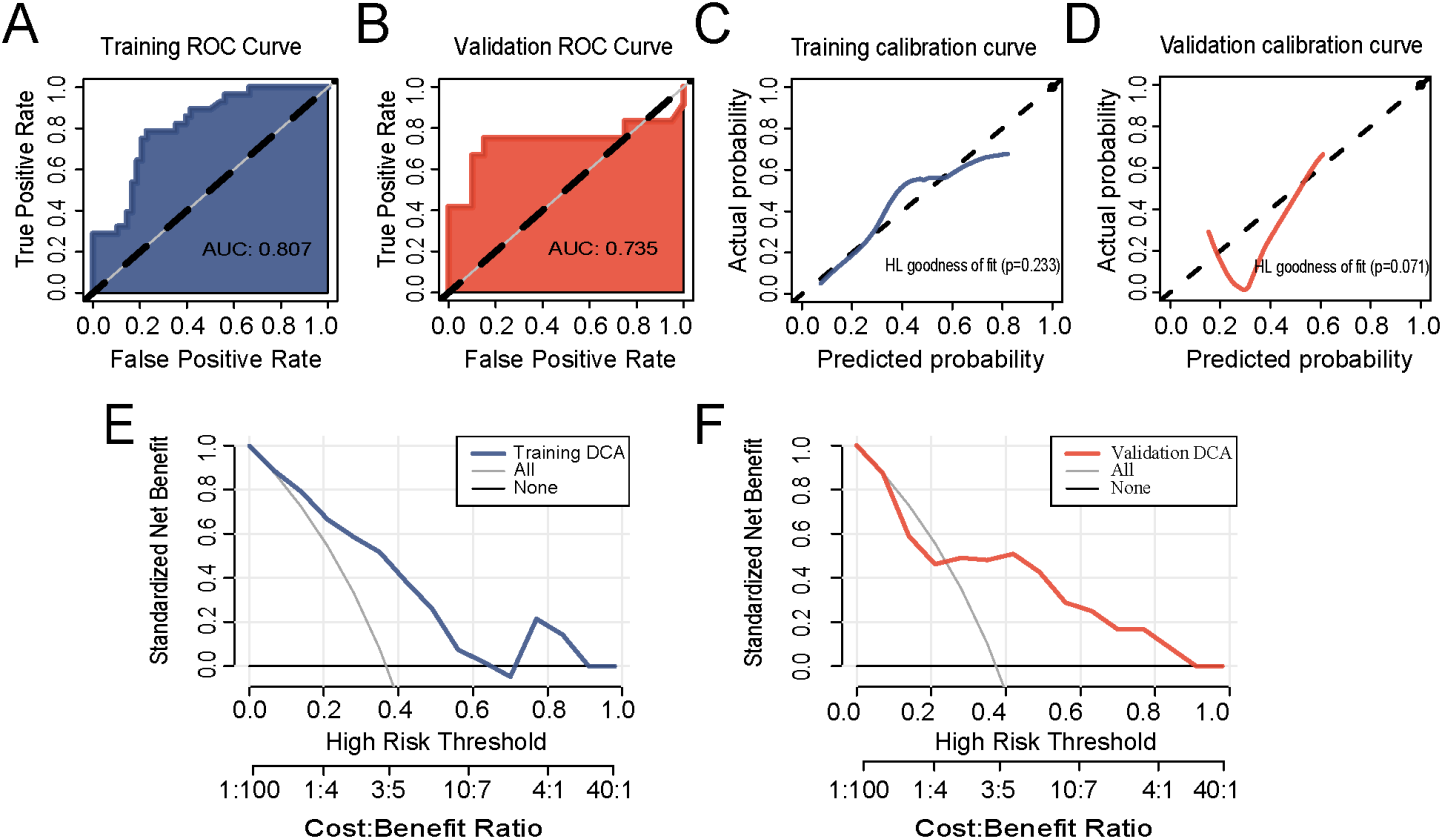
Evaluation of the efficacy of the pathomics model to predict the Treg infiltration level. **(A, B)** Receiver operating characteristic (ROC) curve in the training and validation sets. **(C, D)** Calibration curves and Hosmer–Lemeshow goodness-of-fit test in the training and validation sets. **(E, F)** Decision curve analysis in the training and validation sets.

ROC analysis revealed that the pathomics model exhibited an AUC of 0.807 and 0.735 in the training and validation sets, respectively, indicating that the model had good predictive value (**Figures 2A, B**). The calibration curves (**Figures 2C, D**) along with the Hosmer–Lemeshow goodness-of-fit test (0.233 and 0.071, respectively) indicated a favorable concordance between the predicted probability and the actual level of Treg infiltration in the pathomics model (*P* > 0.05). Based on the DCA, the model exhibited strong clinical applicability (**Figures 2E, F**).

### 3.4 Prognostic relevance of the PS for patients with IDH-wt GBM based on TCGA cohort

The cut-off point for the PS was determined to be 0.299 using the SurvMisc package. This cut-off was derived from the TCGA discovery cohort in an exploratory fashion to enable independent validation. The 108 patients of TCGA cohort were then divided into two groups according to high (n = 65) and low (n = 43) PS values. The distributions of age, sex, KPS score, and chemotherapy did not show significant differences between the high and low PS groups (*P* > 0.05), as indicated in **Table 2**.

**Table 2:** Characteristics of patients in the high and low PS groups in TCGA cohort.

Variables	Total (n = 108)	Low (n = 43)	High (n = 65)	P value
Age, n				0.636
< 60 years	56 (52)	24 (56)	32 (49)	
≥ 60 years	52 (48)	19 (44)	33 (51)	
Gender, n				0.057
Female	38 (35)	10 (23)	28 (43)	
Male	70 (65)	33 (77)	37 (57)	
KPS, n				0.057
< 80	22 (20)	8 (19)	14 (22)	
≥ 80	59 (55)	29 (67)	30 (46)	
Unknown	27 (25)	6 (14)	21 (32)	
MGMT_promoter, n				0.247
Methylated	33 (31)	10 (23)	23 (35)	
Unknown	25 (23)	13 (30)	12 (18)	
Unmethylated	50 (46)	20 (47)	30 (46)	
TERT_promoter, n				0.323
Mutant	40 (37)	13 (30)	27 (42)	
Unknown/WT	68 (63)	30 (70)	38 (58)	
Chr_7_gain_Chr_10_loss, n				0.148
Gain chr 7 & loss chr 10	82 (76)	29 (67)	53 (82)	
No combined CNA	26 (24)	14 (33)	12 (18)	
Radiation, n				0.024
NO	20 (19)	3 (7)	17 (26)	
YES	88 (81)	40 (93)	48 (74)	
Chemotherapy, n				1
NO	31 (29)	12 (28)	19 (29)	
YES	77 (71)	31 (72)	46 (71)	

**Figure 3:**
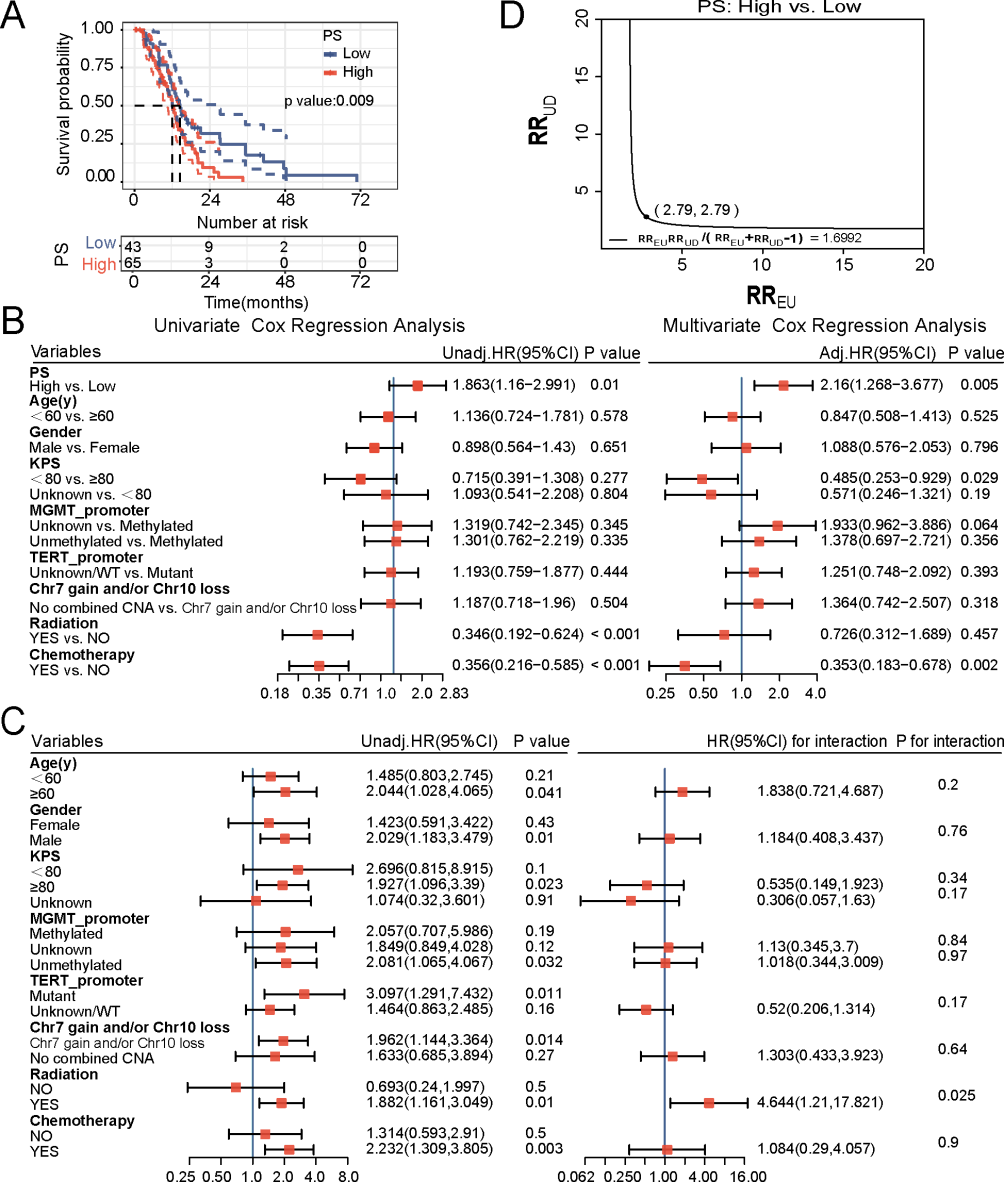
Prognostic relevance of the pathomics score (PS) based on TCGA cohort. **(A)** Kaplan–Meier curve. **(B)** Univariate and multivariate Cox regression analyses. **(C)** Subgroup analysis and interaction tests. **(D)** E-value analysis.

The median survival time for the high and low PS groups was 12 and 14.4 months, respectively, with patients in the high PS group showing significantly worse OS than did those in the low PS group (*P* = 0.009) (**Figure 3A**). This was confirmed in univariate Cox regression analysis, as the high PS group exhibited a poorer prognosis than did the low group (HR = 1.863, 95% CI = 1.160–2.991; *P* = 0.01). In multivariate Cox regression analysis, after adjusting for covariates (Age, gender, KPS, Chr +7/-10), PS remained an independent significant risk factor for OS of patients with IDH-wt GBM (HR = 1.989, 95% CI = 1.182–3.345; *P* = 0.01, **Supplementary Figure 2**). Additionally, to demonstrate the robustness of the association between PS and OS, we further conducted a sensitivity analysis. Firstly, in the multivariate analysis, we included additional confounding factors (Age, gender, KPS, MGMT promoter methylation, TERT promoter mutation, Chr +7/-10, radiation, chemotherapy). After multivariate Cox regression analysis, PS remained an independent prognostic factor for OS, with an HR of 2.160 (1.268-3.677, *P* = 0.005; **Figure 3B**). Subsequently, we further assessed the impact of unknown confounding using the E-value method. The results suggested that the PS-OS association could only be negated by an unmeasured confounder with an exposure-outcome association of at least 2.79 (**Figure 3C**).

The subgroup analysis indicated that elevated PS was a risk factor for OS in the cohort receiving chemotherapy (HR = 2.232, 95% CI = 1.309–3.805; *P* = 0.003; **Figure 3C**) as well as in the cohort receiving radiotherapy (HR = 1.882, 95% CI = 1.161–3.049; *P* = 0.01; **Figure 3D**). Furthermore, the interaction test suggested a significant interaction between PS and radiotherapy status (*P* = 0.025).

### 3.5 Prognostic relevance of PS in IDH-wt GBM patients: evidence from the FJMU cohort

A total of 120 patients who met the inclusion and exclusion criteria underwent the TCGA cohort data processing. This study involved segmenting postoperative pathological HE images, extracting features, and constructing a model to obtain PS values. Using the PS value derived from the TCGA training cohort, patients in the FJMU validation cohort were categorized into 70 cases in the high PS group and 50 in the low PS group. **Table 3** shows no significant differences in age, gender and receipt of chemotherapy between the high and low PS groups (*P* > 0.05).

**Table 3:** Characteristics of patients in the high and low PS groups in FJMU cohort.

Variables	Total (n = 120)	Low (n = 50)	High (n = 70)	P value
Age, n (%)				0.73
< 60	71 (59)	31 (62)	40 (57)	
≥ 60	49 (41)	19 (38)	30 (43)	
Gender, n (%)				0.921
Female	39 (32)	17 (34)	22 (31)	
Male	81 (68)	33 (66)	48 (69)	
MGMT_promoter, n (%)				0.161
Methylated	53 (44)	17 (34)	36 (51)	
Unknown	8 (7)	4 (8)	4 (6)	
Unmethylated	59 (49)	29 (58)	30 (43)	
TERT_promoter, n (%)				0.218
Mutant	43 (36)	18 (36)	25 (36)	
Unknown	20 (17)	5 (10)	15 (21)	
WT	57 (48)	27 (54)	30 (43)	
Chemotherapy, n (%)				0.51
NO	2 (2)	0 (0)	2 (3)	
Unknown	1 (1)	0 (0)	1 (1)	
YES	117 (98)	50 (100)	67 (96)	
EOR, n (%)				0.812
NO	42 (35)	16 (32)	26 (37)	
Unknown	3 (2)	1 (2)	2 (3)	
YES	75 (62)	33 (66)	42 (60)	
OS.time, Median (Q1,Q3)	14.83 (10.57, 23.62)	14.77 (10.84, 26.89)	14.88 (10.29, 21.87)	0.589
OS, n (%)				0.003
0	47 (39)	28 (56)	19 (27)	
1	73 (61)	22 (44)	51 (73)	

**Figure 4:**
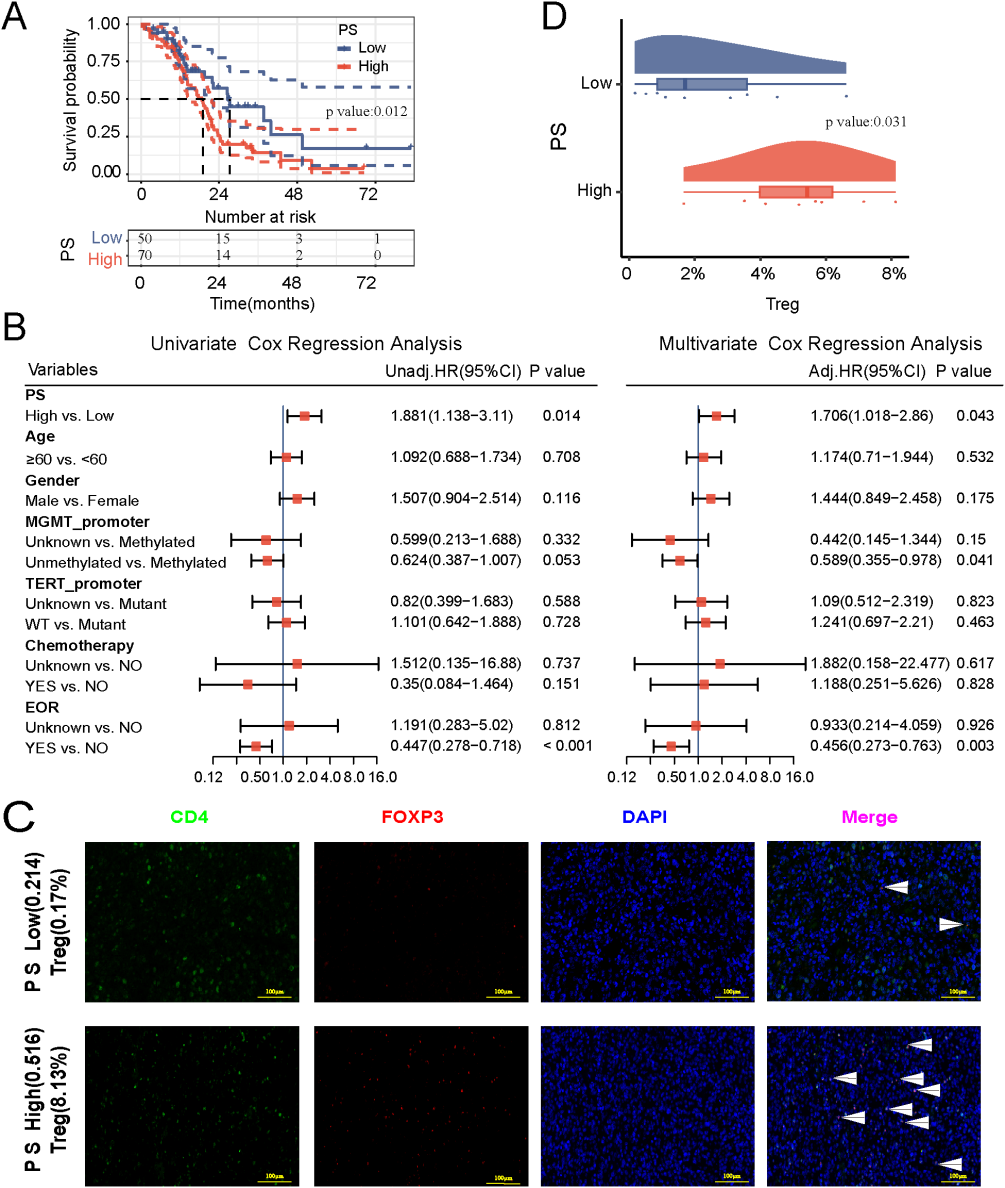
Verify the prognostic relevance of pathomics score (PS) based on the FJMU cohort. **(A)** Kaplan–Meier curve. **(B)** Univariate and multivariate Cox regression analyses. **(C)** Immunofluorescence map of Treg infiltration in brain tissue (green fluorescence: CD4^+^; red fluorescence: FOXP3^+^; blue fluorescence: nucleus; scale: 100 μm). **(D)** Differential analysis of Treg infiltration between the high and low PS groups.

The KM curve demonstrated a significantly lower OS in the high PS group compared to the low PS group (*P =* 0.012, **Figure 4A**), with median survival times of 19 and 27.3 months, respectively. In univariate Cox regression analysis, the PS model effectively differentiated patients with worse and better OS in the FJMU cohorts receiving radiotherapy (HR = 1.881, 95% CI = 1.138-3.110; *P* = 0.014, **Figure 4B**). Extent of resection (EOR) served as a protective factor for OS in GBM patients (HR = 0.447, 95%CI = 0.278-0.718; *P* < 0.001, **Figure 4B**). Multivariate COX analysis confirmed PS as an independent predictor of poor prognosis in GBM patients (**Figure 4B**). Immunofluorescence results indicated a higher level of Treg infiltration in the PS-high group compared to the PS-low group (**Figure 4C**). Differential analysis of the high and low PS subgroups revealed that Treg infiltration was significantly higher in the high PS group compared to the low PS group (*P* = 0.031, **Figure 4D**).

### 3.6 PS-related biological processes

**Figure 5:**
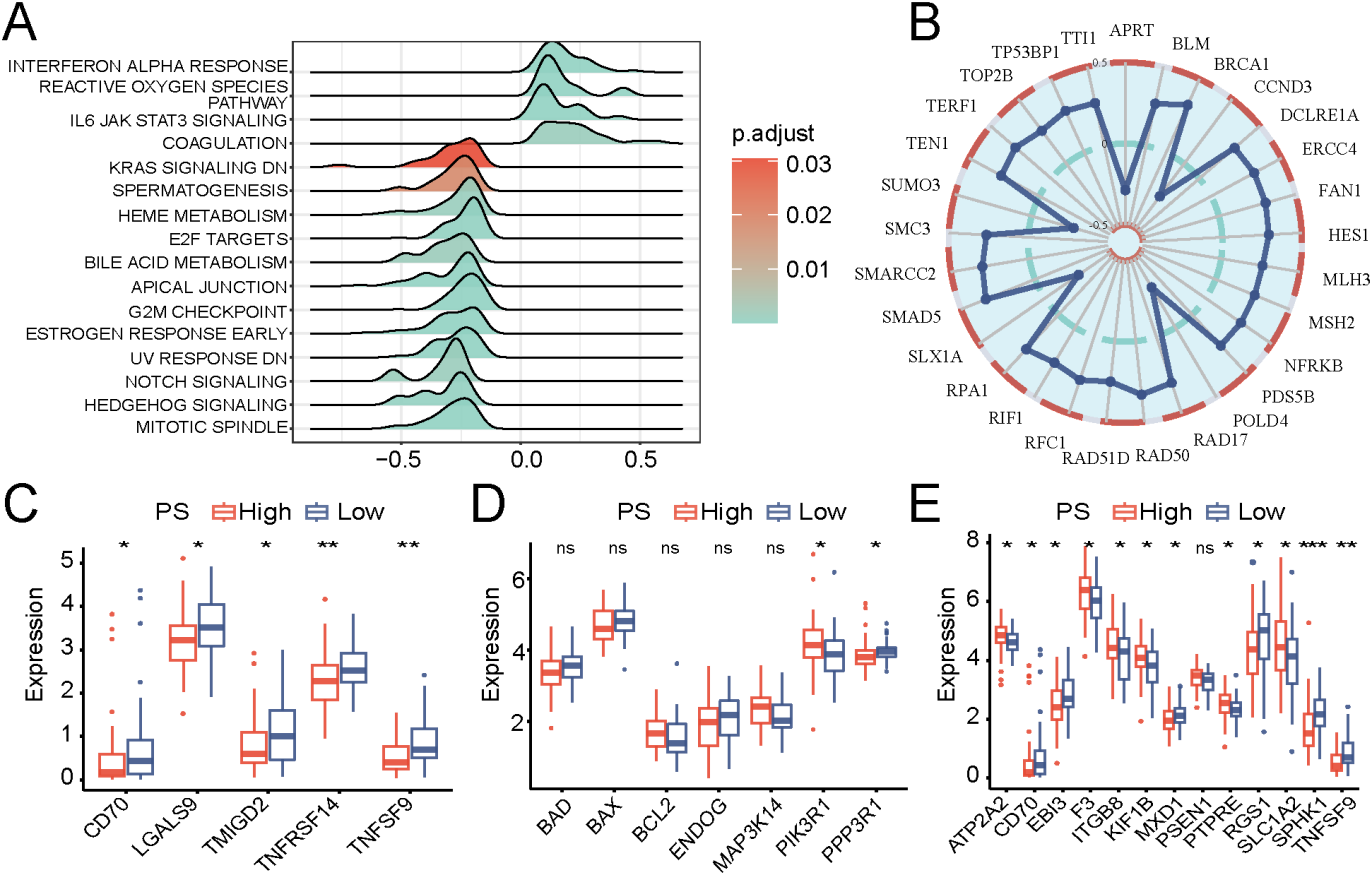
Biological processes associated with the pathomics models. **(A)** Hallmark gene set. **(B)** Radar plot of correlation analysis of the expression levels of genes associated with the pathomics score (PS) and DNA damage repair. **(C)** Differential expression analysis of immune checkpoint-related genes in high and low PS groups. **(D)** Differential expression analysis of apoptosis-related genes in high and low PS groups. **(E)** Differential expression analysis of inflammation-related genes in high and low PS groups. *P < 0.05, **P < 0.01, ***P < 0.001.

Hallmark GSEA revealed significant enrichment of differentially expressed genes in various signaling pathways, including the Notch, IL-6/JAK/STAT3, and heme metabolism pathways (**Figure 5A**). Significant and positive correlations (*P* < 0.05) were observed between the PS and the expression levels of the DNA damage repair-related genes *RAD50*, *BRCA1*, and *ERCC4* (**Figure 5B**). The expression level of the immune checkpoint-related gene *TMIGD2* was significantly lower in the high PS group than in the low PS group (**Figure 5C**, *P* < 0.05).

Furthermore, the high PS group exhibited notably elevated expression levels of the apoptosis-related gene *PIK3R1* and the inflammation-related gene *ITGB8* compared with those of the low PS group (**Figures 5D, E**; both *P* < 0.05).

## 4 Discussion

This AI-based pathomics framework enables stratified treatment planning and may guide clinical decisions on combining radiotherapy with immunotherapeutic interventions targeting Treg-mediated resistance. Owing to the heterogeneity of GBM and varying patient prognoses, even for cases within the same grading, accurate prognostic prediction is crucial for appropriate risk stratification and patient management. Animal experiments and clinical cohorts have consistently shown that Tregs play a key role in the tumorigenesis and development of GBM (8–10), with greater tumor infiltration of Tregs indicating poor prognosis. Tregs have therefore been proposed as useful prognostic markers for GBM (21–23). In this study, we extracted pathomics features from H&E-stained biopsy sections of patients with IDH-wt GBM and established a machine learning-based Treg infiltration abundance prediction model using three pathomics features, which improves interpretability while maintaining predictive performance. Consistent with previous studies, the model revealed a significant correlation between high Treg cell infiltration and PS values. A high PS reflects an H&E-derived histomorphologic pattern associated with Treg-rich, immunosuppressive tumor microenvironments and adverse clinical outcomes. Univariate and multivariate Cox analyses demonstrated that the PS calculated based on H&E images to predict Treg infiltration abundance could serve as an independent prognostic factor for IDH-wt GBM. Additionally, independently validated cohort data indicated that high PS is also an independent prognostic risk factor for OS in GBM patients undergoing radiotherapy.

Qu et al. (24) reported that a pathomics model built based on a deep-learning algorithm accurately predicted recurrence and survival after liver transplantation for hepatocellular carcinoma. However, this model lacked biological interpretability. In contrast, our study employed a machine-learning algorithm that extracted three pathomics features to develop a pathomics scoring model. We further explored the biological significance of the constructed pathomics model, with a focus on three key biological processes linked to PS: Treg-related signaling pathways, DNA damage repair genes, and immune/inflammation/apoptosis-related genes.

First, GSEA results revealed that the PS was associated with Treg-related signaling pathways, including Notch, IL-6/JAK/STAT3, and heme metabolism pathways, all of which are closely involved in Treg cell differentiation, proliferation, and apoptosis (25, 26). These pathways have also been implicated in the progression of various cancers, including GBM, T-cell acute lymphoblastic leukemia, and non-small cell lung cancer (27, 28), supporting our conclusion that PS is mainly associated with Treg infiltration. Specifically, Notch synergizes with TGF-β1 to promote the expression of the Treg-specific transcription factor Foxp3 (29), and the Notch ligand DLL4 further enhances Foxp3 expression and maintains the phenotype and function of induced Tregs (30, 31). Meanwhile, JAK2 and AG490 inhibitors have been shown to improve the phenotype of mice with collagen-induced arthritis by promoting the proportion of Foxp3^+^ Tregs and increasing the expression of molecules associated with Treg development (ICOS, PD-1, ICAM-1, and CD103) (32) while also inhibiting osteoclast development and function.

Additionally, upregulation of heme oxygenase-1 in animal models of allergic asthma and inflammatory bowel disease promotes CD4^+^Foxp3^+^ Treg cell production, exerting an anti-inflammatory effect (33, 34). Given the enrichment of these Treg-related pathways in high PS patients, our model may help identify candidates for clinical trials involving pathway-specific inhibitors or Treg-depleting therapies (e.g., anti-CD25, anti-CTLA4).

Second, we found that the PS value was significantly and positively correlated with the expression levels of DNA damage repair-related genes (*RAD50*, *BRCA1*, and *ERCC4)*.RAD50, a DNA damage repair protein, is upregulated by increased Treg numbers in patients with filarial infection (35). In breast cancer, *RAD50* overexpression confers resistance to various anticancer drugs (36), and elevated *RAD50* expression correlates with aggressive progression of prostate cancer, leading to unfavorable survival outcomes. Similarly, elevated *BRCA1* and *ERCC4* expression has been associated with poor prognosis in several malignant tumors (37, 38). The positive correlation between PS and these genes suggests that high PS (and thus high Treg infiltration) may be associated with enhanced DNA repair capacity, which could contribute to radiotherapy resistance in IDH-wt GBM.

Third, we observed differential expression of immune checkpoint, inflammation, and apoptosis-related genes between high and low PS group: the immune checkpoint gene TMIGD2 was significantly downregulated in the high PS group, while the apoptosis-related gene PIK3R1 and inflammation-related gene ITGB8 were upregulated. TMIGD2, a specific receptor for HHLA2, is predominantly expressed in naïve T cells and natural killer cells, and its binding to HHLA2 stimulates T cell proliferation and activation (39). Increased TMIGD2 expression has been identified as an independent prognostic indicator of favorable outcomes in gliomas (40) and pancreatic cancer (41). In contrast PIK3R1 is involved in cell proliferation, survival, and motility, and its upregulation correlates with immune cell infiltration in most tumors and prognosis in hepatocellular (42, 43). ITGB8, a member of the integrin-chain family, activates TGF-β in keratin-forming cells, and its expression is associated with poor prognosis in various tumors. Yang et al. (44) suggested that *ITGB8* promotes the formation of microvascular and invasive phenotypes in GBM cells by activating the TGFβ1/p-Smad/RhoA signaling pathway, contributing to the poor prognosis of GBM. Furthermore, it has been shown that the absence of the ITGB8 gene in DC cells reduces the proportion of Tregs in mouse colon tissues (45). Other studies have shown that ITGB8 is a key molecule in the differentiation of Th17 cells to Tregs (46). Collectively, these differential gene expressions further support the link between high PS, high Treg infiltration, and poor tumor prognosis, highlighting potential targets for immunotherapy.

Previous studies have demonstrated that targeting Treg inhibition in various tumors, including breast, colorectal, and head and neck cancers, in combination with radiotherapy, enhances radiosensitivity (47–49) and inhibits radiotherapy resistance. Kesarwani et al (50) discovered that the IDO inhibitor GDC-0919 significantly increased the sensitivity of GBM radiotherapy by altering tryptophan metabolism, reducing Treg infiltration levels, and alleviating tumor immunosuppression. Ayman et al. (51) showed that mice with Tregs removed via anti-CD25 treatment had enhanced T-cell toxicity, significantly delayed tumor growth, and prolonged survival time following radiotherapy compared to those of the radiotherapy-alone group, suggesting that Treg removal enhances the therapeutic effect of radiotherapy. Similarly, another study demonstrated that inhibition of the CCR6–CCL20 axis prevented Treg infiltration and sensitized tumors to radiation therapy (49). This study’s results suggest an interaction between Treg, the key component of PS, and radiotherapy. The influence of PS on patient prognosis differed markedly between those receiving radiotherapy and those who did not. Specifically, in the radiotherapy group, PS had a substantial effect on overall survival (OS) with an effect size of 1.882 (*P* = 0.01), compared to its insignificant impact in the non-radiotherapy group (HR = 0.693, *P* = 0.5). This implies that PS is more prognostically relevant in patients undergoing radiotherapy and may be associated with differences in radiotherapy response, which could guide clinical treatment of GBM patients. In an independent external validation cohort receiving radiotherapy, a similar effect was observed (HR = 1.706, *P* = 0.043). Patients with high PS demonstrated significantly reduced OS compared to those with low PS.

Although this exploratory study presents new ideas for future individualized GBM treatments, some limitations need to be addressed. First, this study is retrospective in nature, and prospective validation in larger cohorts is warranted. Second, despite standardizing the data and images in this study, it is necessary to evaluate the potential impact of varying scanning instruments through a unified and forward-looking normalization process. This is due to the whole slides being sourced from multiple institutions, resulting in an imbalance. Additionally, the combination of manual and automatic image segmentation makes the process relatively complex and time-consuming; therefore, optimization is required to improve the efficiency for clinical practice. In addition, given that the vast majority of patients received chemotherapy and only a small percentage did not, we did not include information on TMZ chemotherapy in the baseline data table. And most patients may have received TMZ oral adjuvant chemotherapy at multiple hospitals. Due to the challenges of cross-institutional access within the Chinese health system, we could only obtain chemotherapy data from our hospital. Including only this data would introduce information bias, so we did not include details on chemotherapy cycles or cumulative dosage. Additionally, although we used pathologist-guided random sampling to minimize bias, analyzing only ten sub-images per slide may not fully capture GBM’s spatial heterogeneity; whole-slide or multi-region sampling is warranted in future studies. Finally, the explanatory aspects of the model can only suggest candidate PS-related biological processes, necessitating further *in vivo* and *in vitro* experiments to explore their relevant functions.

## Data Availability

The original contributions presented in the study are included in the article/**Supplementary Material**. Further inquiries can be directed to the corresponding authors.
