# Supplementary figures and images for: AI-based pathomics model predicts regulatory T cell infiltration and radiotherapy response in IDH-wild-type glioblastoma
Source: Front Immunol. 2026 May 21;17:1817892. doi: 10.3389/fimmu.2026.1817892 (PMC13233443; doi:10.3389/fimmu.2026.1817892)

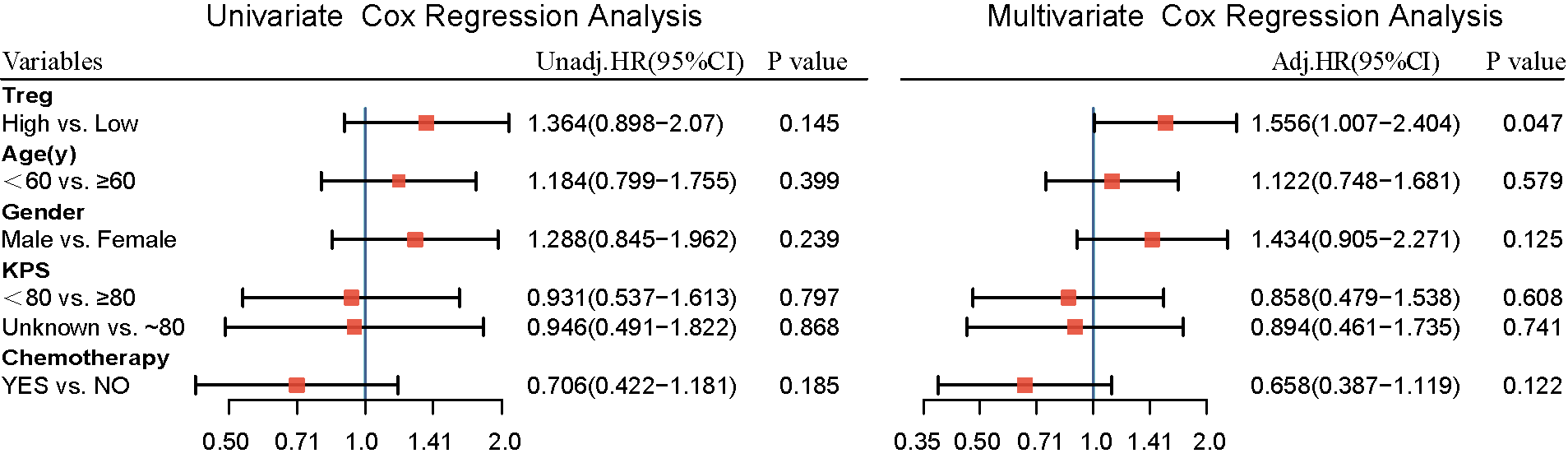

Supplement: Supplementary file 1 [file Image1.tif]

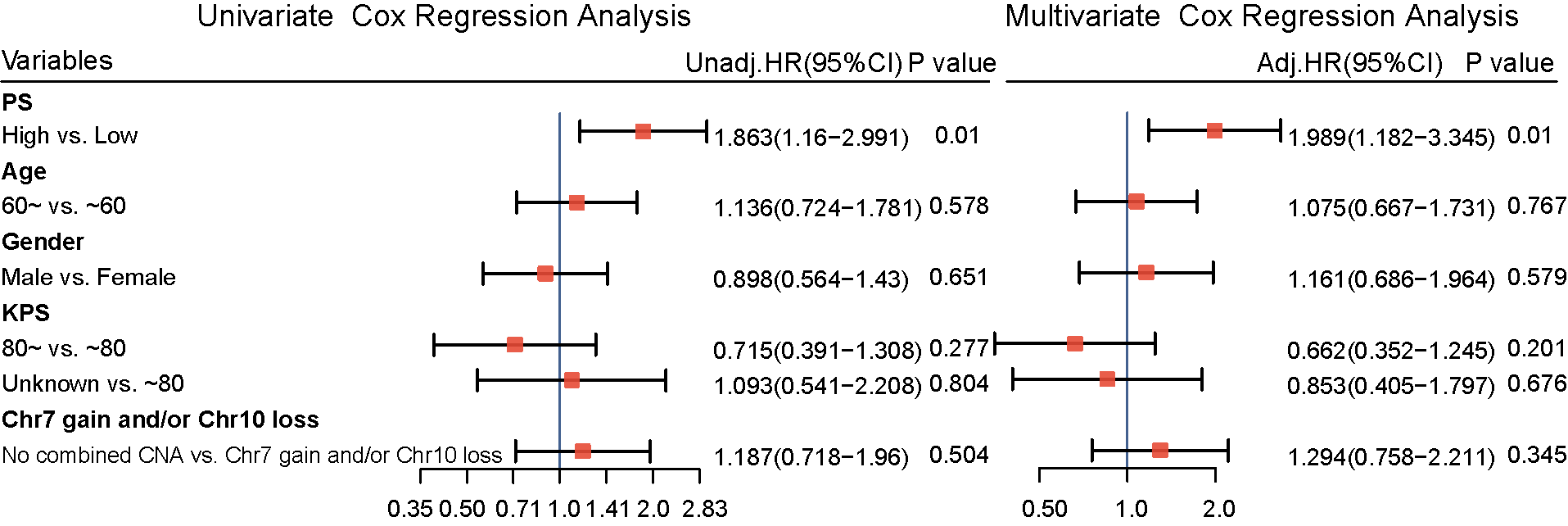

Supplement: Supplementary file 2 [file Image2.tif]
